# Spontaneous Splenic Rupture Associated With Cytomegalovirus Infection: A Case Report

**DOI:** 10.7759/cureus.111978

**Published:** 2026-07-03

**Authors:** Kiara Sejfullai, Federica Giannini, Martina Sorrentino

**Affiliations:** 1 General Surgery, Università Campus Bio-Medico, Rome, ITA; 2 General Surgery, Campus Bio Medico University Hospital, Rome, ITA; 3 General Surgery, Casa di Cura Sant'Anna - Policlinico Città di Pomezia, Pomezia, ITA

**Keywords:** cytomegalovirus, non-traumatic splenic injury, splenectomy, splenic infarction, spontaneous splenic rupture

## Abstract

Atraumatic splenic rupture is a rare but potentially life-threatening condition, most commonly associated with infectious, hematological, neoplastic, inflammatory, or iatrogenic disorders. Cytomegalovirus (CMV)-associated splenic rupture is an exceptionally uncommon complication in immunocompetent individuals. We report the case of a 46-year-old immunocompetent man who presented to the emergency department with acute abdominal pain and a low-grade fever. Laboratory investigations revealed mild microcytic anemia, thrombocytopenia, elevated inflammatory markers, and mild transaminase elevation. Contrast-enhanced abdominal computed tomography demonstrated splenomegaly, a large perisplenic collection consistent with a subcapsular hematoma, and moderate hemoperitoneum, raising suspicion of atraumatic splenic rupture. During clinical observation, the patient developed hemodynamic instability, prompting emergency exploratory laparotomy. Intraoperative findings included massive hemoperitoneum, capsular laceration involving the upper pole, and an extensive subcapsular hematoma. Splenectomy was performed. Postoperative serological investigations revealed markedly elevated anti-CMV IgM levels associated with low anti-CMV IgG levels and low IgG avidity, consistent with acute CMV infection. The pathological examination supported the diagnosis of CMV-associated atraumatic splenic rupture. No antiviral therapy was deemed necessary following infectious disease consultation, and the patient recovered uneventfully with planned post-splenectomy vaccination. CMV infection has increasingly been linked to thrombotic and vascular complications, even in immunocompetent individuals. Proposed mechanisms of splenic rupture include endothelial dysfunction, transient prothrombotic states, lymphocytic infiltration of splenic tissue, ischemic injury, and reduced capsular elasticity. Since clinical manifestations are often nonspecific and may mimic other causes of acute abdomen, diagnosis can be challenging. Contrast-enhanced computed tomography remains the gold standard for diagnosis and therapeutic planning. Management depends on the severity of splenic injury and the patient's hemodynamic status; however, splenectomy remains the preferred treatment in cases of severe injury or hemodynamic deterioration. This case highlights the importance of considering CMV infection in the differential diagnosis of atraumatic splenic rupture and underscores the value of early imaging and prompt intervention in optimizing patient outcomes.

## Introduction

Spleen rupture is predominantly associated with blunt abdominal trauma, while non-traumatic ruptures represent a rare condition, usually arising from infectious diseases or hematological or oncological disorders. Spontaneous splenic rupture represents a critical diagnostic and therapeutic emergency, requiring prompt diagnosis and management. The first documented case of cytomegalovirus (CMV)-related splenic infarction was reported by Jordan et al. in 1973 [1]. Initially considered an uncommon occurrence in immunocompetent individuals, recent literature has noted an increase in reported cases of splenic infarction linked to CMV infections [2]. Splenic rupture represents a possible complication that, although infrequent, can lead to significant morbidity and mortality. This article presents a case of spontaneous splenic rupture in an immunocompetent patient due to acute CMV infection, contributing to the evidence on this critical yet understudied association.

## Case presentation

**Figure 1:**
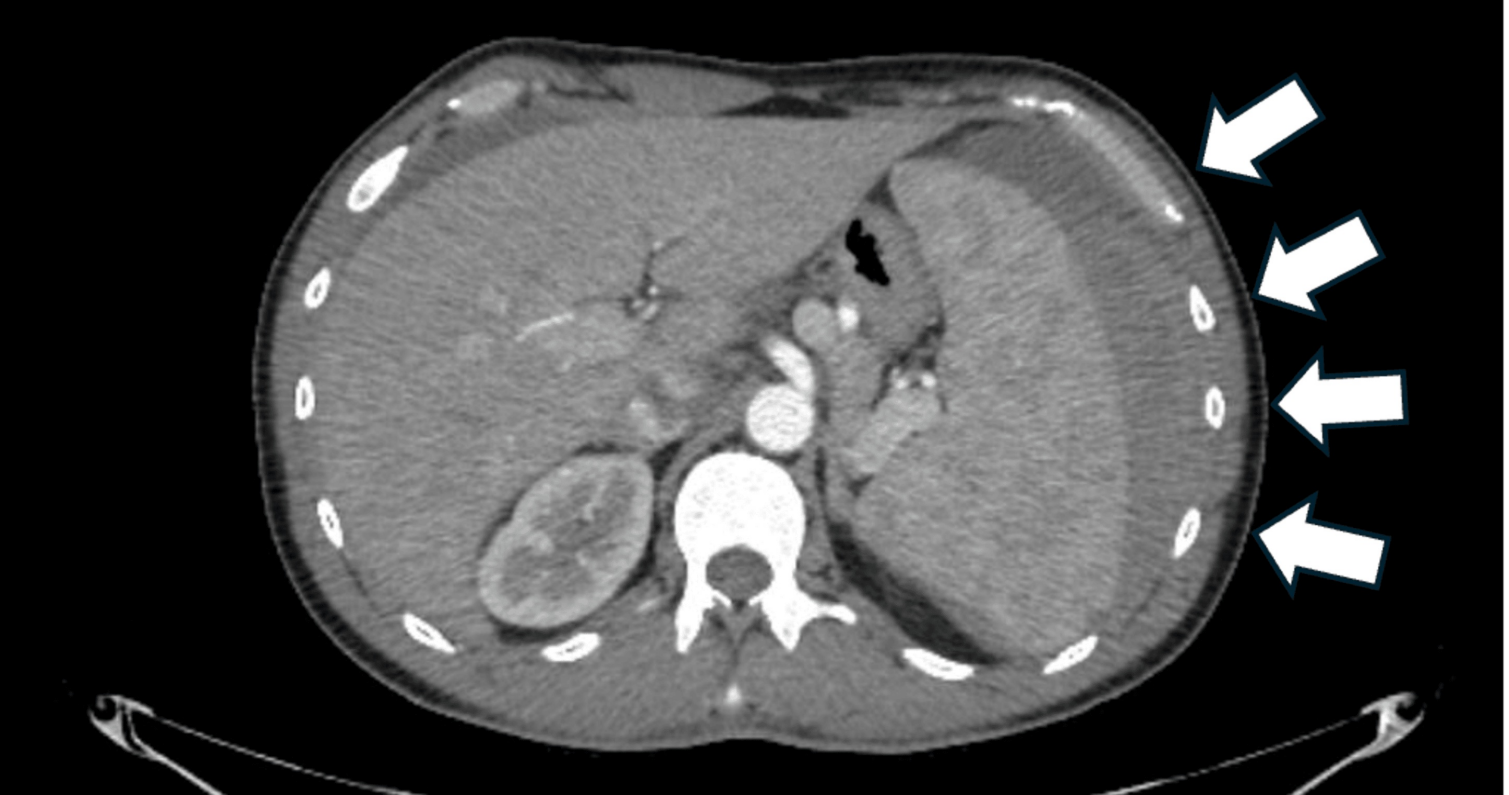
Abdominal CT scan showing splenomegaly, subcapsular hematoma, and hemoperitoneum

A 46-year-old man with no significant past medical history presented to the emergency department of Casa di Cura Sant’Anna Policlinico Città di Pomezia with acute abdominal pain. The patient reported episodes of fever (<38.5°C) during the previous weeks. On admission, the patient was hemodynamically stable. Initial laboratory investigations demonstrated mild microcytic anemia, with a hemoglobin level of 10.7 g/dL, a white blood cell count of 8.3 × 10³/mm³ (neutrophils 56.8%, lymphocytes 30.3%, monocytes 9.2%, eosinophils 1.1%, basophils 2.6%), and mild thrombocytopenia, with a platelet count of 133 × 10³/mm³. C-reactive protein (CRP) was mildly elevated (1.5 mg/dL). Liver function tests revealed mild transaminase elevation without serological evidence of viral hepatitis or biochemical signs of cholestatic liver injury (AST 53 U/L, ALT 43 U/L, gamma-glutamyl transferase 23 U/L, total bilirubin 0.72 mg/dL, direct bilirubin 0.14 mg/dL, indirect bilirubin 0.58 mg/dL). Serum lipase was within the normal range (38 U/L), whereas serum amylase was mildly elevated (173 U/L). Contrast-enhanced abdominal computed tomography (CT) demonstrated splenomegaly, with a longitudinal splenic diameter of approximately 15 cm, associated with a perisplenic collection showing mixed hyperdense and hypodense components, suggestive of a subcapsular hematoma with possible active bleeding (Figure 1). A moderate amount of free intraperitoneal fluid, predominantly within the pelvic cavity, was also identified. The extent of the subcapsular hematoma, involving more than 50% of the splenic surface area, was consistent with a Grade III splenic injury according to the American Association for the Surgery of Trauma (AAST) Organ Injury Scale.

Based on the clinical and radiological findings, atraumatic splenic rupture was suspected. During clinical observation, approximately 90 minutes after presentation to the emergency department, the patient developed signs of circulatory compromise, reduction in urine output (<25 mL/h), and progressive hypotension (blood pressure 80/50 mmHg), leading to hemodynamic instability. Emergency surgical intervention was the treatment of choice, as interventional radiology was not available at our institution. Furthermore, the patient's progressive hemodynamic instability precluded safe transfer to a nearby referral center with interventional radiology capabilities, making surgery the only viable therapeutic option. As soon as the patient’s clinical condition deteriorated, he was taken to the operating theater for an emergency laparotomy. Intraoperative exploration revealed massive hemoperitoneum. The spleen appeared markedly enlarged, with a capsular laceration involving the upper pole and a large subcapsular hematoma extending throughout nearly the entire splenic parenchyma. Laparotomic splenectomy was performed.

During the postoperative course, serological investigations were negative for SARS-CoV-2 infection and anti-HCV antibodies, while anti-HBs antibodies were reactive. Markedly elevated anti-CMV IgM antibody index (7.8) was detected, associated with low anti-CMV IgG antibody index (<0.4) and low CMV IgG avidity (<40%), consistent with acute CMV infection. Histopathological examination of the surgical specimen revealed an enlarged spleen measuring 18 × 11.5 × 5 cm, with capsular laceration and extensive subcapsular hemorrhagic areas. Microscopic evaluation demonstrated rupture-associated hemorrhage with reactive hyperplasia of the red pulp and focal mild hyperplasia of the white pulp. Immunohistochemical analysis showed no evidence of lymphoproliferative, myeloproliferative, or metastatic neoplastic disease. No Hodgkin/Reed-Sternberg-like cells were identified. Reticulin architecture was preserved, without evidence of fibrosis or extramedullary hematopoiesis. The immunohistochemical specific staining for CMV resulted positive, compatible with the clinical suspicion of atraumatic splenic rupture associated with CMV infection. Overall, the clinical presentation, radiological findings, intraoperative evidence, serological profile, and histopathological results supported the diagnosis of atraumatic splenic rupture associated with acute CMV infection. The patient underwent an infectious disease department consultation, stating that no antiviral therapy was needed. Post-splenectomy vaccinations were programmed after dimission.

## Discussion

Spontaneous splenic rupture represents an uncommon condition compared with the more frequent traumatic rupture. The systematic review proposed by Renzulli et al. detected six major etiological groups for spontaneous splenic rupture: infectious, neoplastic, inflammatory, congenital or structural, iatrogenic, and idiopathic [3]. Regarding the infectious etiology, few case reports in the literature have shown a correlation with CMV. CMV is a DNA virus belonging to the *Herpesviridae* family, with seroprevalence reaching approximately 50% in developed countries [4]. Humans represent the only known reservoir, and most infections are asymptomatic. It has been shown to promote endothelial dysfunction by increasing the expression of endothelial adhesion molecules and von Willebrand factor, thereby contributing to a prothrombotic state. CMV infections have been increasingly associated with thrombotic events. Atzmony et al. reported that acute CMV infection is independently associated with thrombosis, even in the absence of other recognized thrombotic risk factors [5].

The pathophysiology of splenic rupture in CMV infection remains not fully understood. Proposed mechanisms include massive lymphocytic infiltration of the splenic parenchyma and vascular walls, leading to acute ischemic injury, parenchymal fragility, reduced capsular elasticity, and transient hemostatic abnormalities commonly associated with infectious diseases. Protopapa et al. described several mechanisms of vasculopathy and ultimately hypothesized a suppressive effect of CMV on the mechanism of action of protein S [6]. Non-traumatic splenic rupture may occur as either a one-stage or two-stage event. The one-stage rupture presents acutely, rapidly progressing to hemodynamic instability and hemorrhagic shock. In contrast, the two-stage rupture is initially characterized by the formation of a subcapsular hematoma, associated with a more insidious clinical presentation, followed by delayed rupture of the splenic capsule and subsequent hemoperitoneum, as seen in our clinical case [7]. Patients with spontaneous splenic rupture typically manifest signs of hypovolemic shock associated with acute abdominal pain and tenderness in the left upper quadrant. Additionally, they may suffer from nausea, vomiting, syncope, and dizziness. Pain can radiate to the left shoulder, especially in the supine position, due to diaphragmatic irritation by intraperitoneal blood (Kehr’s sign). Clinical presentation may, however, be atypical and mimic other acute conditions [8].

In the described cases in the literature, in CMV splenic rupture, the most common findings include left upper quadrant abdominal pain, signs of peritonitis, and hemorrhagic shock. The absence of prodromal symptoms during acute infection in immunocompetent adults may contribute to delayed diagnosis [9]. Regarding diagnosis, imaging, in addition to the clinical aspect, represents the gold standard. Abdominal ultrasonography (US) and CT are considered the most valuable imaging modalities for spontaneous splenic rupture diagnosis. US may demonstrate splenomegaly, capsular irregularities, and the presence of intraperitoneal fluid. Abdominal CT remains the imaging of choice, allowing definitive identification of splenic injury, subcapsular hematoma, and intra-abdominal fluid collections. Furthermore, it enables accurate grading of splenic rupture, assisting in therapeutic management [8].

In our case, an abdominal CT scan was prioritized to provide high-resolution identification of the injuries, ensuring adequate time-sensitive management. Early recognition and prompt management are essential to reduce morbidity and mortality related to this condition. Treatment strategies depend on the severity of splenic injury and the patient’s hemodynamic status. In hemodynamically stable patients with low-grade splenic injury, conservative management consisting of fluid resuscitation, blood transfusion, when necessary, and close monitoring in an intensive care setting may be appropriate [10]. In cases of severe splenic injury or persistent hemodynamic instability, invasive management may be required, including splenic artery embolization, spleen-preserving surgical procedures, or splenectomy. Overall, approximately 20-40% of patients with atraumatic splenic rupture ultimately require surgical treatment [11]. Regarding medical management, antiviral treatment with Ganciclovir or Valganciclovir has been employed in some cases [8,9]; however, in our case, an infectious disease consultation determined that no antiviral treatment was indicated. This approach is consistent with Protopapa et al., who emphasized that Ganciclovir is primarily reserved for immunocompromised patients and is just occasionally considered in immunocompetent patients [6].

## Conclusions

Non-traumatic spontaneous splenic rupture remains a rare clinical entity, with CMV-induced etiology representing an even rarer manifestation. Because its initial presentation often mimics other acute abdominal emergencies, early high-resolution diagnostic imaging via contrast-enhanced abdominal CT scan is crucial in order to prevent diagnostic delays and to guide a prompt therapeutic intervention.

Physicians should maintain a suspicion for CMV-related splenic rupture in patients presenting with acute abdominal pain associated with pyrexia and imaging that detects splenic rupture. Anti-CMV IgM and IgG antibody indices as well as CMV IgG avidity should be evaluated in order to have a diagnosis of acute CMV infection, complemented by immunohistochemical evaluation of the surgical specimen, when available. This case report expands the limited literature on this critical yet understudied clinical association.
